# Supplementary material for: Non-hydrolyzable acetyllysine analogs to study protein acetylation in vitro and in cells
Source: Nat Commun. 2026 Feb 21;17:1985. doi: 10.1038/s41467-026-69782-6 (PMC12932645; doi:10.1038/s41467-026-69782-6)
Supplement: Supplementary file 2 — Reporting Summary [file 41467_2026_69782_MOESM2_ESM.pdf]

nature portfolio

Corresponding author(s): Simon Maria Kienle, Martin Scheffner

Last updated by author(s): Jan 31, 2026

Reporting Summary

Nature Portfolio wishes to improve the reproducibility of the work that we publish. This form provides structure for consistency and transparency in reporting. For further information on Nature Portfolio policies, see our Editorial Policies and the Editorial Policy Checklist.

Statistics

For all statistical analyses, confirm that the following items are present in the figure legend, table legend, main text, or Methods section.

n/a	Confirmed	
☐	☒	The exact sample size (n) for each experimental group/condition, given as a discrete number and unit of measurement
☐	☒	A statement on whether measurements were taken from distinct samples or whether the same sample was measured repeatedly
☐	☒	The statistical test(s) used AND whether they are one- or two-sided<br>*Only common tests should be described solely by name; describe more complex techniques in the Methods section.*
☒	☐	A description of all covariates tested
☐	☒	A description of any assumptions or corrections, such as tests of normality and adjustment for multiple comparisons
☐	☒	A full description of the statistical parameters including central tendency (e.g. means) or other basic estimates (e.g. regression coefficient) AND variation (e.g. standard deviation) or associated estimates of uncertainty (e.g. confidence intervals)
☒	☐	For null hypothesis testing, the test statistic (e.g. F, t, r) with confidence intervals, effect sizes, degrees of freedom and P value noted<br>*Give P values as exact values whenever suitable.*
☒	☐	For Bayesian analysis, information on the choice of priors and Markov chain Monte Carlo settings
☒	☐	For hierarchical and complex designs, identification of the appropriate level for tests and full reporting of outcomes
☐	☒	Estimates of effect sizes (e.g. Cohen's d, Pearson's r), indicating how they were calculated

Our web collection on statistics for biologists contains articles on many of the points above.

Software and code

Policy information about availability of computer code

Data collection	All MS/MS data was collected on an QExcactive HF Hybrid Quadrupole-Orbitrap operated with Tune (version 2.9). MS data were collected on a micrOTOF II (Bruker) interfaced with an Agilent 1260 Infinity II liquid chromatography system. NMR experiments were performed at T=298K on an Avance Neo 800 MHz spectrometer equipped with a quadruple (QCI) cryo probe with z-gradient. Gel and Western Blot pictures were taken on an Fujifilm LAS-3000 Imager. Luciferase reporter assays were measured with Wallac 1420 VICTOR³ Multilabel plate reader (PerkinElmer)
Data analysis	MaxQuant (version 1.6.8), Perseus software (version 1.6.10.50), Proteome Discoverer 1.4 , Mascot 2.6, Compass DataAnalysis 4.1 (Bruker), Skyline software 23.1.0.268, NMRPipe (version 10.1), NMRView (version 8.0.a27), Adobe Illustrator CS4, GraphPad Prism 6 (GraphPad Software; version 6.01), AIDA Image Analyzer (raytest), Fiji ImageJ (version 1.51)

For manuscripts utilizing custom algorithms or software that are central to the research but not yet described in published literature, software must be made available to editors and reviewers. We strongly encourage code deposition in a community repository (e.g. GitHub). See the Nature Portfolio guidelines for submitting code & software for further information.

Data

Policy information about availability of data

All manuscripts must include a data availability statement. This statement should provide the following information, where applicable:

- Accession codes, unique identifiers, or web links for publicly available datasets
- A description of any restrictions on data availability
- For clinical datasets or third party data, please ensure that the statement adheres to our policy

The NMR solution structure and the crystal structure of ubiquitin used in this study are available in the ProteinDataBank under the accession codes1D3Z [https://doi.org/10.2210/pdb1D3Z/pdb] and 1UBQ [https://doi.org/10.2210/pdb1UBQ/pdb], respectively. The mass spectrometry proteomics data have been deposited to the ProteomeXchange Consortium (http://proteomecentral.proteomexchange.org) via the PRIDE partner repository with the dataset identifier PXD056063 (Username: reviewer_pxd056063@ebi.ac.uk; Password: tCMcGvYVb79f) for the AE-MS data and PXD056021 (Username: reviewer_pxd056021@ebi.ac.uk; Password: AWE0DQbBpn9D) for the PRM data of the p53 acetylation status. All other data generated in this study are provided in the Supplementary Information and the Source Data file.

Research involving human participants, their data, or biological material

Policy information about studies with human participants or human data. See also policy information about sex, gender (identity/presentation), and sexual orientation and race, ethnicity and racism.

Reporting on sex and gender	Study did not involve human participants, their data, or biological material
Reporting on race, ethnicity, or other socially relevant groupings	n/a; see above
Population characteristics	n/a; see above
Recruitment	n/a; see above
Ethics oversight	n/a; see above

Note that full information on the approval of the study protocol must also be provided in the manuscript.

Field-specific reporting

Please select the one below that is the best fit for your research. If you are not sure, read the appropriate sections before making your selection.

- [x] Life sciences
- [ ] Behavioural & social sciences
- [ ] Ecological, evolutionary & environmental sciences

For a reference copy of the document with all sections, see nature.com/documents/nr-reporting-summary-flat.pdf

Life sciences study design

All studies must disclose on these points even when the disclosure is negative.

Sample size	Sample sizes were not predetermined based on statistical methods, but were chosen according to the standards of the field (at least three independent biological replicates for each condition). AE-MS samples were prepared in three biological experiments for all investigated samples, and each of these was measured with technical duplicates.
Data exclusions	No data was excluded, only search criteria applied as described in the mansucript.
Replication	AE-MS samples were prepared in three biological experiments for all investigated samples, and each of these was measured with technical duplicates.<br>In vitro interaction studies were performed twice.<br>In vitro ubiquitination experiments were performed three times.<br>PRM analysis of the p53 acetylation status was performed three times.<br>Luciferase reporter experiments were performed at least three times.<br>All attempts at replication were successful.
Randomization	No human or animal subjects were used in the study. Randomization is generally not used in the field and not applicable for the approaches used. AE-MS samples were measured by members of the Proteomics Center of the University of Konstanz that apart from that were not involved in the respective experiments.
Blinding	n/a. Blinding was not applicable, as for the analysis of the experiments reaction samples need to be correctly labeled. AE-MS samples were measured by members of the Proteomics Center of the University of Konstanz that apart from that were not involved in the respective experiments. Furthermore, blinding is not used in the field.

Reporting for specific materials, systems and methods

We require information from authors about some types of materials, experimental systems and methods used in many studies. Here, indicate whether each material, system or method listed is relevant to your study. If you are not sure if a list item applies to your research, read the appropriate section before selecting a response.

Materials & experimental systems

n/a	Involved in the study
☐	☒ Antibodies
☐	☒ Eukaryotic cell lines
☒	☐ Palaeontology and archaeology
☒	☐ Animals and other organisms
☒	☐ Clinical data
☒	☐ Dual use research of concern
☒	☐ Plants

Methods

n/a	Involved in the study
☒	☐ ChIP-seq
☒	☐ Flow cytometry
☒	☐ MRI-based neuroimaging

Antibodies

Antibodies used

Primary antibodies:, anti-p21 (#2947, Cell Signalling Technologies), anti-NDP52 (ab68588, abcam), anti-VCP (ab109240, abcam), anti-RAD23A (ab108592, abcam), anti-USP13 (ab109264, abcam), anti-BRCC3 (HPA048737, Atlas Antibodies), anti-XRCC6 (NBP2-34247, Novus biologicals), anti-p53 (DO-1, Calbiochem (cat# OP43, Sigma)), Anti-His (HIS-1, cat# A7058, Sigma Aldrich), anti-HA.11 (16B12, #901515, BioLegend). Primary antibodies were used as 1:1,000 dilutions.
Secondary antibodies: mouse (115-035-062, Jackson ImmunoResearch), rabbit (111-035-003, Jackson ImmunoResearch). Secondary antibodies were used as 1:15,000 dilutions.

Validation

Validation of each primary antibody is available on the manufacturers website.
anti-p21 [https://www.cellsignal.com/products/primary-antibodies/p21-waf1-cip1-12d1-rabbit-monoclonal-antibody/2947]
anti-NDP52 [https://www.abcam.com/en-us/products/primary-antibodies/ndp52-antibody-ab68588]
anti-VCP [https://www.abcam.com/en-us/products/primary-antibodies/vcp-antibody-epr33072-ab109240]
anti-RAD23 [https://www.abcam.com/en-us/products/primary-antibodies/hhr23a-antibody-epr4818-ab108592]
anti-USP13 [hhttps://www.abcam.com/en-us/products/primary-antibodies/usp13-antibody-epr4348-ab109264]
anti-BRCC3 [https://www.atlasantibodies.com/products/primary-antibodies/triple-a-polyclonals/anti-brcc3-antibody-hpa048737/]
anti-XRCC6 [https://www.novusbio.com/products/ku70-xrcc6-antibody-ku729_nbp2-34247]
anti-p53 [https://www.sigmaaldrich.com/DE/en/product/mm/op43]
anti-HIS [https://www.sigmaaldrich.com/DE/en/product/sigma/a7058]
anti-HA.11 [https://www.biolegend.com/en-us/products/anti-ha-11-epitope-tag-antibody-11071]

Eukaryotic cell lines

Policy information about cell lines and Sex and Gender in Research

Cell line source(s)	ATCC (HEK293T, #CRL-3216; H1299, #CRL-5803)
Authentication	The cell lines were not authenticated.
Mycoplasma contamination	Cell lines were not tested for Mycoplasma contamination.
Commonly misidentified lines (See ICLAC register)	No commonly misidentified cell line was used in this study.

Plants

Seed stocks	n/a
Novel plant genotypes	n/a
Authentication	n/a
